# Genetic Susceptibility to Neurodegeneration in Amazon: Apolipoprotein E Genotyping in Vulnerable Populations Exposed to Mercury

**DOI:** 10.3389/fgene.2018.00285

**Published:** 2018-07-27

**Authors:** Gabriela P. F. Arrifano, Rosa C. R. Martín-Doimeadios, María Jiménez-Moreno, Sergio Fernández-Trujillo, Marcus Augusto-Oliveira, José R. Souza-Monteiro, Barbarella M. Macchi, Jacqueline I. Alvarez-Leite, José L. M. do Nascimento, Marcos T. Amador, Sidney Santos, Ândrea Ribeiro-dos-Santos, Liz C. Silva-Pereira, Reinaldo B. Oriá, Maria E. Crespo-Lopez

**Affiliations:** ^1^Laboratório de Farmacologia Molecular, Instituto de Ciências Biológicas, Universidade Federal do Pará, Belém, Brazil; ^2^Department of Analytical Chemistry and Food Technology, Faculty of Environmental Sciences and Biochemistry, University of Castilla-La Mancha, Toledo, Spain; ^3^Laboratório de Investigações em Neurodegeneração e Infecção (Hospital Universitário João de Barros Barreto), Universidade Federal do Pará, Belém, Brazil; ^4^Laboratório de Neuroquímica Molecular e Celular, Instituto de Ciências Biológicas, Universidade Federal do Pará, Belém, Brazil; ^5^Departamento de Bioquímica e Imunologia, Universidade Federal de Minas Gerais, Belo Horizonte, Brazil; ^6^Neuroscience Research Group, CEUMA University, São Luís, Brazil; ^7^Laboratório de Genética Humana e Médica, Instituto de Ciências Biológicas, Universidade Federal do Pará, Belém, Brazil; ^8^Instituto Federal de Educação, Itaituba, Brazil; ^9^Laboratório da Biologia da Cicatrização, Ontogenia e Nutrição de Tecidos, Departamento de Morfologia e Instituto de Biomedicina, Escola de Medicina, Universidade Federal do Ceará, Fortaleza, Brazil

**Keywords:** ApoE, Tapajós, Tucuruí, human, methylmercury, ancestry, allele, genotype

## Abstract

Human exposure to mercury is a serious problem of public health in Amazon. As in other vulnerable populations throughout the world, Amazonian riverine populations are chronically exposed to this metal and some symptoms of mercury intoxication were already detected in these populations. However, studies on the genetic susceptibility to mercury toxicity in the Amazon are scarce, and they tested a limited number of individuals. In this context, apolipoprotein E gene (*APOE*) is a key element with a well-established association among their alleles and the neurodegenerative consequences of mercury intoxication. However, no studies have addressed *APOE* genotyping in Amazonian exposed populations. Additionally, epidemiological studies with *APOE* genotyping in Amazon have been restricted to indigenous populations. Therefore, this work analyzed for the first time the genotypic and allelic profiles of *APOE* in Amazonian riverine populations chronically exposed to mercury. Eight hundred and twenty three individuals were enrolled in our study donating blood (794) and/or hair (757). *APOE* genotyping was analyzed by real-time PCR. Total mercury and mercury species were quantified by ICP-MS and GC-pyro-AFS, respectively. Genomic ancestry markers were evaluated by multiplex-PCR reaction, separated by capillary electrophoresis on the ABI 3130 Genetic Analyzer instrument and analyzed on GeneMapper ID v3.2. The 𝜀3 and 𝜀3/𝜀3 were the most frequent allele and genotype, respectively, followed by 𝜀4 allele and 𝜀3/𝜀4 genotype. Only 𝜀2/𝜀2 genotype was not found, suggesting that the absence of this genotype is a generalized phenomenon in Amazon. Also, our data supported an association between the presence of *APOE4* and the Amerindian origin in these populations. Fifty-nine individuals were identified at maximum risk with levels of mercury above 10 μg/g and the presence of *APOE4*. Interestingly, among individuals with high mercury content, *APOE4*-carriers had high mercury levels than *APOE2*-carriers, pointing to a different heavy metal accumulation according to the *APOE* allele. These data suggest that *APOE4*, in addition to a possible pharmacodynamic effect, may influence pharmacokinetically the mercury exposure causing its higher accumulation and leading to worse deleterious consequences. Our results may aid in the development of prevention strategies and health policy decision-making regarding these at-risk vulnerable populations.

## Introduction

Human exposure to mercury is presently a major public health concern worldwide (Sheehan et al., 2014; Andreoli and Sprovieri, 2017). Since 2013, a growing number of countries are agreeing to join efforts aiming to protect human health and the environment against the deleterious effects of this heavy metal. The result of the conceptualization of this collaborative platform was the Minamata Convention on Mercury^1^, endorsed by the World Health Organization^2^ (WHO) and ratified by Brazil on August, 2017.

Brazil is a continent-sized country that faces huge challenges in the consolidation of an universal healthcare system (Coutinho and Silva Junior, 2015; Muzaka, 2017). The northern region of Brazil is almost entirely occupied by the Amazon rainforest, where human populations have been suffering from mercury exposure since the 1980s, when the “the gold rush” occurred (Berzas Nevado et al., 2010). According to the WHO, tropical populations near gold mining locations show the highest weekly mercury intake among all vulnerable populations in the world, reaching almost four times the FAO/WHO reference level of 2.2 μg/g (Sheehan et al., 2014). In addition to gold mining activities, other anthropogenic alterations of the Amazonian environment such as hydroelectric power dams may be contributing to release the mercury containing in soils, facilitating the methylation of the metal, and making it available for human exposure (Marques et al., 2011; Hacon et al., 2014; Fearnside, 2015; Arrifano et al., 2018b).

The main human populations affected by mercury exposure in Amazon are remote communities with two different profiles, also known as indigenous and riverine people. The indigenous populations are native organized into tribes who are direct descendants of the original populations that lived in Amazon before the European colonization. Brazilian laws recognize and protect these populations, presently living at protected lands (reservoirs). The riverine populations are rural communities located at riverbanks, with high genetic introgression due to colonization and immigration and with no special protection by Brazilian laws. Both populations share some characteristics, such as economy of subsistence, fish as the main protein of the diet and no sanitary conditions or piped water (Piperata, 2007). Also, both of them live in remote areas with difficult access to health centers (da Silva Souza et al., 2018).

Worryingly, several studies have demonstrated high mercury content in hair of some riverine populations in Amazon (Berzas Nevado et al., 2010; Marques et al., 2013; Hacon et al., 2014; Hoshino et al., 2015; Arrifano et al., 2018b). These communities are exposed mainly to methylmercury (MeHg) through consumption of contaminated fish (Berzas Nevado et al., 2010; Rodriguez Martin-Doimeadios et al., 2014; Arrifano et al., 2018b), as other vulnerable populations throughout the world (Drescher et al., 2014; Strain et al., 2015; Langeland et al., 2017; Salazar-Camacho et al., 2017). MeHg is the most toxic specie of mercury with diverse effects, such as neurotoxicity and genotoxicity among others (Crespo-Lopez et al., 2011, 2016; Farina et al., 2011). Still, the neurotoxic effects of MeHg are recognized as the most deleterious consequences in humans (Farina et al., 2011; Arrifano et al., 2018a). MeHg intoxication can produce several neurological effects, such as motor and visual impairment, mood change and memory loss (Grandjean et al., 1999; Dolbec et al., 2000; Rodrigues et al., 2007; Fillion et al., 2011a,b; Khoury et al., 2015; Costa et al., 2017; Da Silva-Junior et al., 2017). The neurodegeneration caused by this metal was already associated with the development of neurodegenerative diseases such as Alzheimer’s and Parkinson’s (Farina et al., 2013; Chin-Chan et al., 2015). Despite this scenario and the importance of developing adequate prevention strategies, studies on the genetic susceptibility to mercury toxicity in the Amazon are scarce, and they tested a limited number of individuals (Klautau-Guimarães et al., 2005; Jacob-Ferreira et al., 2010, 2011; Barcelos et al., 2013, 2015; de Oliveira et al., 2014; Rocha et al., 2016), probably because the difficulties to reach these communities and the demanding conditions to carry out these studies.

Although some markers of genetic susceptibility to mercury toxicity have been proposed in recent years (reviewed by Andreoli and Sprovieri, 2017), apolipoprotein E gene (*APOE* for the gene; and ApoE for the protein) is a key element in this scenario, with a well-established association among their alleles and the neurodegenerative consequences (Godfrey et al., 2003; Wojcik et al., 2006; Ng et al., 2013, 2015; Woods et al., 2014; Snoj Tratnik et al., 2017). ApoE is a glycoprotein involved in several brain functions, including lipid metabolism, axonal growth, synaptic formation and neuronal repair (Xu et al., 2014; Arrifano et al., 2018a). *APOE* is a polymorphic gene with three common alleles, 𝜀2, 𝜀3 and 𝜀4, which differ in the arginine and cysteine contents at positions 112 and 158 of the protein (Mahley et al., 2009; Arrifano et al., 2018a). ApoE3 shows a cysteine and an arginine at positions 112 and 158, respectively, whereas ApoE2 has two cysteines and ApoE4 has two arginines at both positions (Mahley et al., 2009). The presence of *APOE4* is the only genetic risk factor confirmed in the development of late-onset Alzheimer Disease (Bertram, 2009; Xu et al., 2014) and it is presently the gene with more studies demonstrating an association with the susceptibility to mercury-induced neurotoxicity in adults (Andreoli and Sprovieri, 2017; Arrifano et al., 2018a).

Therefore, this work analyzed, for the first time in Amazon, the apolipoprotein E genotyping in riverine populations chronically exposed to mercury. The possible associations with ancestry and exposure were also studied.

## Materials and Methods

### Participants

**FIGURE 1:**
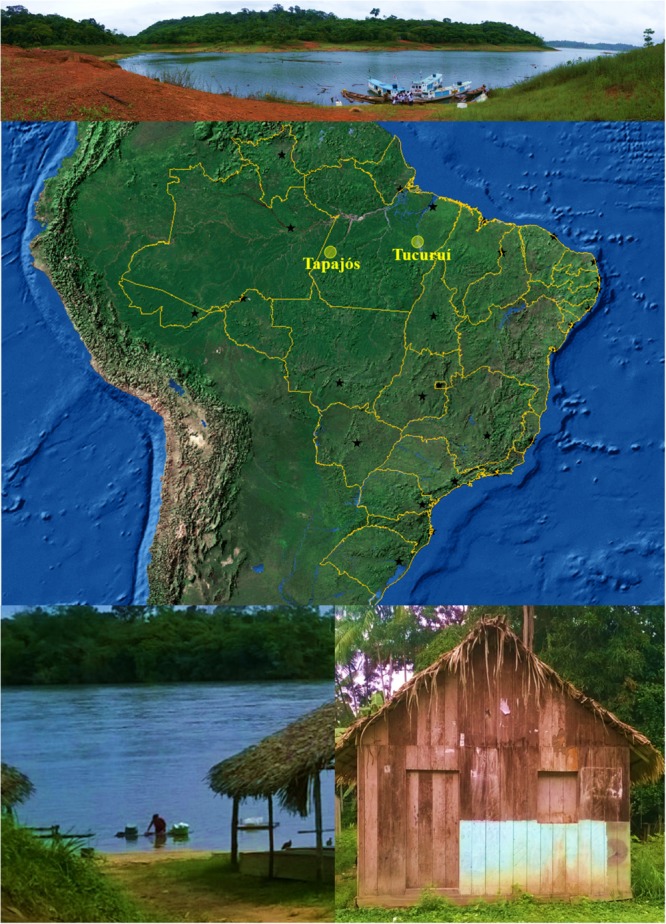
Map of Brazil (obtained from the Instituto Brasileiro de Geografia e Estatística, Brazil) showing the States and their capitals (*black stars*). *Yellow circles* indicate the locations (–4.287121, –55.984106, and –3.800897, –49.811848) of riverine populations participating of this study. Photographs illustrates some conditions of the riverine populations: difficult access (above), river as a key element (*below left*) and precarious facilities (*below right*).

Amazonian riverine populations chronically exposed to mercury via consumption of contaminated fish were included in the present work (**Figure 1**). Participants were from two locations (Tapajós River basin and Tucuruí Lake) that were demonstrably exposed to mercury in the past and in the present (Berzas Nevado et al., 2010; Rodriguez Martin-Doimeadios et al., 2014; Khoury et al., 2015; Arrifano et al., 2018b) and included adults (≥18 years) living in the area for at least 2 years.

The present study followed the guidelines established by both the Declaration of Helsinki and the Conselho Nacional de Ética em Pesquisa com Seres Humanos (CONEP, Brazil) and it was approved with the CAAE no. 43927115.4.0000.0018. All participants gave written consent to participate after understanding the purpose of this work.

### Anthropometric Data and Self-Reported Conditions

Gender, age, fish-intake and the existence of drug dependency (including tobacco and alcohol), pre-existent chronic diseases and chronic pharmaceutical treatments were registered for each participant. Also, weight and height were measured.

### Sample Collection

Approximately, 0.1 g of hair from the occipital region (1–2 cm from the scalp) with clean stainless scissors and 2 mL of blood were drawn and stored (in paper envelopes at room temperature and frozen in EDTA vacutainer tubes, respectively).

### DNA Extraction

Genomic DNA was isolated from whole blood with the Purelink Genomic DNA Mini Kit (ThermoFisher, Brazil), according to manufactures’ instructions. Quantitation of DNA was performed using the Qubit dsDNA BR Assay Kit on a Qubit 3.0 fluorimeter (ThermoFisher, Brazil).

### Apolipoprotein E Genotyping

*APOE* genotype of each participant was detected by real time-PCR with TaqMan^®^ assays using the StepOnePlus^®^ equipment (ThermoFisher, Brazil). Two *APOE* single-nucleotide polymorphisms (SNPs), rs429358 and rs7412, corresponding to the positions 112 and 158 of the protein, respectively, were analyzed. For each SNP the reactions were performed in duplicate on 96-well microtiter plates with final volume of 10 μl, containing 5 μl of 2× TaqMan Universal PCR Master Mix (ThermoFisher, Brazil), 25 nmol/l of each probe (FAM or VIC-labeled), and 0.5 μl of DNA (30–100 ng). The run conditions were 40 cycles of denaturation at 92°C for 15 s and hybridization and extension at 60°C for 1 m. Each SNP’s probes had two sequences labeled with a different dye VIC/FAM to the polymorphisms C/T, respectively. This technique permits the identification of the six possible genotypes: 𝜀2/𝜀2; 𝜀2/𝜀3; 𝜀2/𝜀4; 𝜀3/𝜀3; 𝜀3/𝜀4; and 𝜀4/𝜀4, according the combination of fluorescence detected for each position. So, 𝜀2 had only the detection of FAM fluorescence; 𝜀3 had FAM for rs429358 and VIC for rs7412 fluorescence detection and; 𝜀4 had only VIC detection for both SNPs.

### Ancestry

Genomic ancestry analysis was performed based on the method described elsewhere (Ramos et al., 2016), using 61 autosomal ancestry informative markers (AIMs). Two multiplex PCR reactions of 20 and 22 markers were performed and amplicons were analyzed by electrophoresis using the ABI Prism 3130 sequencer and GeneMapper ID v.3.2 software. The individual proportions of European, African, and Amerindian genetic ancestries were estimated using STRUCTURE v.2.3.3 software, assuming three parental populations (European, African, and Amerindian).

### Quantification of Total Mercury and Methylmercury in Human Hair

Extraction of Hg species was performed in a closed-vessel microwave oven using 100 mg of hair sample and 10 mL of 6 N nitric acid as extractant. Microwave vessels were sealed and irradiated for 5 min at 80°C after a 5 min ramping time. A clear solution was obtained after microwave irradiation. Extraction blanks were also prepared in the same manner in each batch.

Total mercury content in hair samples were determined by inductively coupled plasma mass spectrometry (ICP-MS, Thermo Fisher Corporation) and mercury species were determined by GC-pyro-AFS system according to Arrifano et al. (2018b). For total mercury analysis, extracts were directly analyzed after the adequate dilution. For mercury species determination, 2 mL of the acidic extracts were subjected to derivatization with sodium tetraethylborate and mercury species were extracted in hexane. For derivatization, the pH of the extracts was adjusted to 3.9 using ammonia (30%) and 5 mL of 0.1 M acetic acid-sodium acetate buffer. Then, 2 mL of hexane and 500 μL of (3%, w/v) were added and the mixture was manually shaken for 5 min. The sample was centrifugated for 5 min at 600 *g*. The organic layer was transferred to a chromatographic glass vial and stored at -18°C until analysis. The certified reference material human hair ERM-DB001 (Sigma-Aldrich, Brazil) was used for quality control of mercury analysis.

### Statistical Analysis

Gaussian distribution of data was verified with D’Agostino-Pearson normality test. Accordingly, data were then analyzed with Mann–Whitney test to compare groups. Correlations between variables were tested by Spearman test. Distributions and frequencies were evaluated with Chi-square or Fisher’s exact tests. Hardy–Weinberg equilibrium (HWE) was tested to verify whether *APOE* genetic variation remained at equilibrium. For all analysis, *p* < 0.05 was considered statistically significant.

## Results

Anthropometric data of the participants of the present study are showed in **Table 1**. Except for the distribution of gender, no significant difference was detected for all anthropometric parameters between participants from the two regions.

**Table 1:** Anthropometric profile of the participants of the study.

	Total *n* = 823 (100%)	Tapajós *n* = 466 (56.62%)	Tucuruí *n* = 357 (43.38%)	*p*-Value Tapajós vs. Tucuruí
Gender (% female)	63.3	66.3	59.4	<0.05
Age (y)	47 (33–57)	47 (32–59)	47 (35–56)	0.324
Height (m)	155 (151–163)	155 (151–162)	155 (151–163)	0.523
Weight (kg)	64.5 (56.2–74.8)	64.2 (56.0–74.2)	65.0 (57.0–75.6)	0.242
BMI (kg/m^2^)	26.2 (23.4–29.7)	26.2 (23.3–29.6)	26.2 (23.4–29.8)	0.680

*Data are presented as median and interquartile ranges. All parameters were analyzed with Mann–Whitney test except the distributions of gender that were evaluated with Fisher’s exact test. BMI, body mass index (calculated as weight in kilograms divided by the square of height in meters).*

**FIGURE 2:**
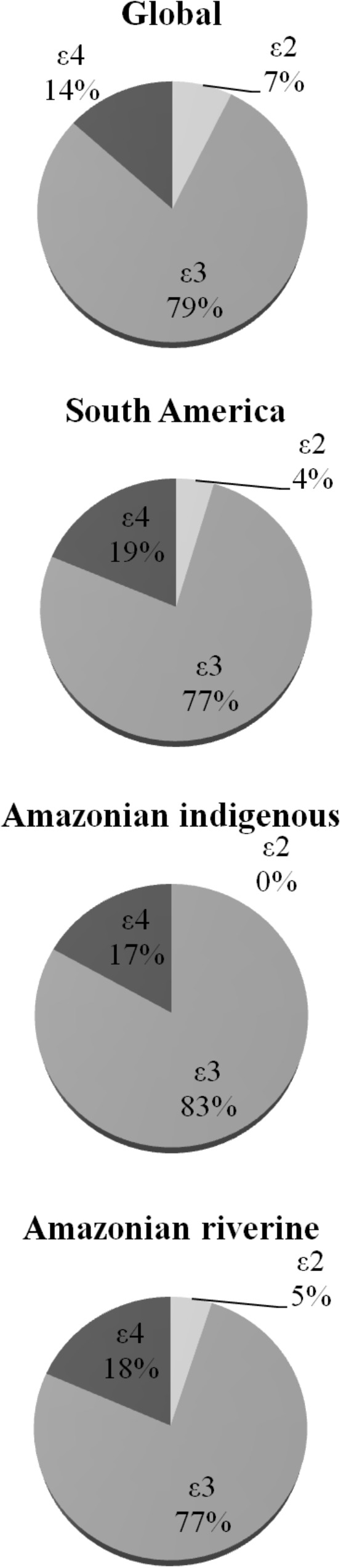
Allelic distributions of apolipoprotein E gene in global (mainly developed countries), South American, Amazonian indigenous, and Amazonian riverine populations. Data are from Singh et al. (2006) and this work.

**Figure 2, Table 2**, and Supplementary Table S1 includes the genotypic and allelic frequencies of apolipoprotein E found in the participants. Only the 𝜀2/𝜀2 genotype of *APOE* gene was not found. No difference was detected between participants from Tapajós and Tucuruí. For the following analyses with *APOE4*-carriers or non-carriers, the individuals with the 𝜀2/𝜀4 genotype (14 participants) were not included since both alleles may show opposite effects on mercury intoxication.

**Table 2:** Genotypic and allelic distributions and frequencies (*f*) of apolipoprotein E in riverine populations of the Amazon.

	Total		Tapajós		Tucuruí	
Genotypes	*n*	(%)	*n*	(%)	*n*	(%)
						
𝜀2/𝜀2	0	(0.0)	0	(0.0)	0	(0.0)
𝜀2/𝜀3	65	(8.2)	38	(8.5)	27	(7.8)
𝜀2/𝜀4	14	(1.8)	9	(2.0)	5	(1.4)
𝜀3/𝜀3	468	(58.9)	272	(61.0)	196	(56.3)
𝜀3/𝜀4	215	(27.1)	116	(26.0)	99	(28.5)
𝜀4/𝜀4	32	(4.0)	11	(2.5)	21	(6.0)
Total	794	(100.0)	446	(100.0)	348	(100.0)
HWE, *p*-value	0.602		0.902		0.463	
**Allele frequency**	***f***	***f (%)***	***f***	***f (%)***	***f***	***f (%)***
						
𝜀2	0.050	5.0	0.053	5.3	0.046	4.6
𝜀3	0.766	76.6	0.783	78.3	0.744	74.4
𝜀4	0.185	18.5	0.165	16.5	0.210	21.0

*No difference was found between participants from Tapajós River basin and Lake of Tucuruí (Chi-square test, *p* > 0.05). HWE, Hardy–Weinberg equilibrium.*

Ancestry markers revealed a significant high contribution of European origin, followed by Amerindian and by African origins (**Table 3**) (Kruskal–Wallis test, *p* < 0.0001). No difference in ancestry distribution was detected between participants from Tapajós and Tucuruí.

**Table 3:** Ancestry profile of the riverine populations of Amazon.

Ancestry	Total *n* = 794	Tapajós *n* = 446	Tucuruí *n* = 348	*p-*Value Tapajós vs. Tucuruí
European (%)	42.4 (32.7–51.1)	42.9 (31.7–53.1)	41.7 (33.5–49.2)	0.255
Amerindian (%)	31.9 (22.6–41.7)	30.5 (21.3–42.5)	32.4 (25.1–41.1)	0.174
African (%)	22.9 (16.4–30.7)	22.9 (17.2–30.4)	22.6 (15.8–31.6)	0.800

*Data are presented as median and interquartile ranges. No difference was found between participants from Tapajós River basin and Lake of Tucuruí (Mann–Whitney test).*

Considering that no difference was found between Tapajós and Tucuruí in both distributions of ancestry and apolipoprotein E genotypes and alleles, we studied the possible association of the presence of ApoE4 and ancestry for the total population. *APOE4*-carriers (𝜀4/𝜀4 and 𝜀3/𝜀4) showed a significant higher proportion of Amerindian origin when compared to that of non-carriers (**Table 4**).

**Table 4:** Ancestry profile of the Amazonian riverine individuals according to be apolipoprotein 𝜀4 allele (*APOE4*) carrier or not.

Ancestry	*APOE4*-carrier *n* = 247	*APOE4* non-carrier *n* = 533	*p-*Value
European (%)	42.4 (32.1–50.4)	42.3 (33.2–51.4)	0.494
Amerindian (%)	33.4 (25.6–43.5)	31.3 (22.0–40.8)	<0.01
African (%)	22.2 (15.8–29.9)	23.2 (16.5–31.7)	0.066

*Data are presented as median and interquartile ranges and they were analyzed with the Mann–Whitney test.*

To confirm the present exposure of the populations, we quantified the mercury content in hair of participants. Seven hundred and fifty-seven participants agreed to donate a hair sample for mercury quantification. Total mercury level in hair was 4.84 μg/g (2.30–9.66) as median and interquartile ranges, being 87.8% (83.7–90.3) MeHg. Mercury levels were correlated with the frequency of fish intake (number of meals per week) (Spearman test, *p* < 0.05). There was no correlation of age with total mercury concentrations (Spearman test, *p* > 0.05). However, men showed significantly higher mercury levels than women, consuming also higher amounts of fish (Mann–Whitney test, *p* < 0.001). Moreover, the median level of mercury was significantly higher in Tucuruí (8.12, 3.65–14.99 μg/g) than in Tapajós (3.62, 1.77–6.47 μg/g) (Mann–Whitney test, *p* < 0.001), therefore, the following analyses with mercury levels were carried out for three universes of sampling (the total of participants, those from Tapajós and those from Tucuruí) to reveal any difference between locations.

No significant difference was found in allelic frequencies of *APOE* between individuals with high and low mercury levels (**Table 5A**). These results were observed even when rigorous exclusion criteria were additionally applied (which are not always present in studies of human exposure), guaranteeing the elimination of most confounding factors and the potential for mercury levels to be significantly influenced by altered hepatic and/or renal function (**Table 5B**).

**Table 5:** Allelic frequencies of apolipoprotein E gene in participants with total mercury levels above (High Hg) and below (Low Hg) the limit of 10 μg/g.

Allelic distribution	Total			Tapajós			Tucuruí		
(A) No exclusion criterion									
	Low Hg *n* = 550	High Hg *n* = 193	*p*-Value	Low Hg *n* = 367	High Hg *n* = 54	*p*-Value	Low Hg *n* = 183	High Hg *n* = 139	*p*-Value
𝜀2	0.05	0.04	0.430	0.05	0.06	0.305	0.06	0.03	0.673
𝜀3	0.76	0.77		0.77	0.81		0.75	0.76	
𝜀4	0.18	0.19		0.18	0.12		0.20	0.22	
**(B) With rigorous exclusion criteria**									
	**Low Hg *n* = 345**	**High Hg *n* = 120**	***p*-Value**	**Low Hg *n* = 230**	**High Hg *n* = 33**	***p*-Value**	**Low Hg *n* = 115**	**High Hg *n* = 87**	***p*-Value**

𝜀2	0.04	0.04	0.973	0.04	0.09	0.126	0.04	0.02	0.478
𝜀3	0.77	0.77		0.76	0.76		0.78	0.78	
𝜀4	0.19	0.19		0.20	0.15		0.18	0.20	

*To consider the participants for the analyses, no exclusion criterion **(A)** or rigorous exclusion criteria (older age >70 years), drug dependency (including tobacco and alcohol), the presence of pre-existent (chronic diseases and chronic pharmaceutical treatments) **(B)**, were applied. Data were analyzed with Chi-square test.*

**FIGURE 3:**
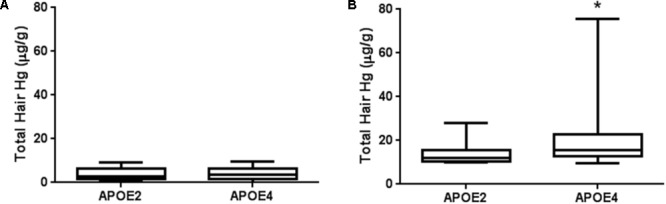
Total mercury levels in hair of *APOE2*-carriers and *APOE4*-carriers in Amazonian riverine population. Individuals with 𝜀2/𝜀4 genotypes were not included in the present analysis. **(A)** Participants with mercury levels below 10 μg/g (*n* = 47 and 172 for *APOE2*- and *APOE4*-carriers, respectively); **(B)** Participants with mercury levels ≥10 μg/g (*n* = 12 and 59 for *APOE2*- and *APOE4*-carriers, respectively). Data are presented as median and interquartile ranges. ^∗^*p* < 0.05. Mann–Whitney test.

Interestingly, when *APOE2*-carriers (𝜀2/𝜀3) were compared to *APOE4*-carriers, mercury levels of the latter ones were significantly higher in individuals with high mercury content (**Figure 3**). It is important to note that both groups included similar proportions of men and women and consumed similar amounts and frequencies of fish meals (**Table 6**). These results already point to a different mercury bioaccumulation affected by *APOE* alleles in high mercury participants. This difference was not detected in individuals with mercury levels below the limit (**Figure 3**).

**Table 6:** Anthropometric profile and fish consumption (frequency and amount) in *APOE2*- and *APOE4*-carriers with high levels of mercury (≥10 μg/g).

	*APOE2*-carriers *n* = 12	*APOE4*-carriers *n* = 54	*p*-Value
Gender (% females)	41.7%	49.2%	0.756^a^
Age (y)	42 ± 4.2	45 ± 1.8	0.347^b^
BMI (kg/m^2^)	25.74 ± 1.033	26.04 ± 0.5964	0.831^b^
Fish consumption			
Weekly frequency, number of meals	6 (3–14)	5 (3–7)	0.751^c^
Amount of fish per meal, grams	300 (160–400)	300 (170–390)	0.913^c^

*Individuals with 𝜀2/𝜀4 genotypes were not included in the present analysis. Data are presented as mean ± SD or median and interquartile ranges according to Gaussian distribution. ^*a*^Fisher’s exact test. ^*b*^Student’s *t*-test. ^*c*^Mann–Whitney test.*

## Discussion

This work showed for the first time the genotypic and allelic distribution of apolipoprotein E in Amazonian riverine populations and its association with ancestry. Additionally, our data revealed that individuals exposed to high mercury levels showed a more Amerindian ancestral background. Interestingly, a higher accumulation of mercury was detected in *APOE4*-carriers when compared to *APOE2*-carriers.

Amazon is the largest tropical forest in the world, most of the area belonging to Brazil. Currently, Brazilian Amazon is occupied by more than 17 million people (IBGE, 2016), being one of the huge challenges in healthcare that faces this developing country with an emerging economy (Muzaka, 2017). Amazonian populations show the lowest human development index (Atlas of Human Development in Brazil, 2013) with a significant part of the population living far away from the main cities and organized in small riverine communities or widespread family based houses. Presently, Amazonian riverine populations has a particular life style with the river as a central element (**Figure 1**): water for cooking is obtained from the river or hand-dug wells, fish is the main protein of the diet (usually included in many meals per week) and trash is sometimes dumped into the river (Piperata, 2007). A different number of women and men participated in our study (**Table 1**), which is usually found in epidemiological studies with Amazonian population (Krewer et al., 2011; Valentini et al., 2016). The hypothesis raised to explain this difference is that, in these communities, women may be more careful with health than men may (Gomes et al., 2007). Interestingly, in Tucuruí participation of men was as high as that of women (**Table 1**), probably because men commanded the boats with all the family to the collection point (personal observation). Notably, of the initial 823 participants that attended to the collection point, only 29 individuals abstained to donate blood (or the collection was not possible), contributing for the representability of the sample.

To our knowledge, this is the larger epidemiological study of mercury-related genetic factors carried out in Amazonian riverine population (Klautau-Guimarães et al., 2005; Jacob-Ferreira et al., 2010, 2011; Barcelos et al., 2013, 2015; de Oliveira et al., 2014; Rocha et al., 2016). Additionally, studies genotyping *APOE* gene are really scarce in Amazonian populations and they are limited to small indigenous tribes, such as Yanomami or Tsiname (Crews et al., 1993; Marin et al., 1997; Jaramillo-Correa et al., 2001; Trumble et al., 2017).

Our results demonstrated that 𝜀3 and 𝜀3/𝜀3 were the most frequent allele and genotype, respectively (**Table 2**), followed by 𝜀4 allele and 𝜀3/𝜀4 genotype. Other interesting result is despite of the large number of participants, the 𝜀2/𝜀2 genotype was not detected (**Table 2**). Previous studies with smaller samples of participants from Amazonian indigenous tribes (Crews et al., 1993; Marin et al., 1997; Jaramillo-Correa et al., 2001; Trumble et al., 2017) did not find this genotype either (**Figure 2**). Based on our data with a large number of participants, the absence of the 𝜀2/𝜀2 genotype would not be restricted to the indigenous profile in Amazon, suggesting that it could be a general phenomenon in Amazonian populations.

When compared to other populations around the world (**Figure 2**), we observed a profile of allelic and genotypic frequencies in Amazonian riverine populations, similar to that found in other populations of South-America, characterized by a higher frequency of 𝜀4 allele and a lower frequency of 𝜀2 allele (Singh et al., 2006). In fact, approximately 33% of the participants of the present study carried at least one copy of the 𝜀4 allele (**Table 2**).

The 𝜀4 allele is considered the ancestral one in the genus *Homo*, which would have been progressively lost due to the high risk involving the development of neurodegeneration and cardiovascular diseases (Singh et al., 2006; Eisenberg et al., 2010; Trumble et al., 2017). Hypothesis were already raised to explain its higher prevalence in developing countries or traditional societies, such as an apparent increased resistance to pathogens and/or a rebalancing in cholesterol levels in conditions of increased metabolic rate (which would partially compensate the increased risk of neurodegeneration) (Corbo and Scacchi, 1999; Mahley and Rall, 1999; Singh et al., 2006; Mahley et al., 2009; Eisenberg et al., 2010; Trumble et al., 2017). The possible association with Amerindian origin make us to analyze the ancestral background of the participants of the study.

Historically, the colonization by the Europeans lead to a high genetic exchange of the Amazonian population (Cavalcante et al., 2015). Brazilian population shows a trihybrid ancestry with three major contributors (Amerindian, European, and African) (Parra et al., 2003; Lins et al., 2010). In most of the Brazilian regions, the European origin provides the highest contribution (Lins et al., 2010). Amazonian riverine population also shows this profile with 42.4% of European ancestry (**Table 3**). In this population, Amerindian contribution was significantly higher than African. Interestingly, *APOE4*-carriers showed a significant higher Amerindian contribution than that of *APOE4* non-carriers (**Table 4**), suggesting a possible association between the two factors. Considering that in the Amazon, the Amerindian profile may be associate to primitive societies, our results would be in agreement with the idea that the 𝜀4 allele remains significantly prevalent in economies of foraging and/or with food restrictions (Corbo and Scacchi, 1999) and/or when metabolic rate is elevated due to the high energetic expenditure on cooling/thermogenesis (for example Eisenberg et al., 2010). In this case, *APOE4*-carriers would show an adaptive advantage because the higher cholesterol absorption and body burden when compared to those of 𝜀3/𝜀3 individuals (Corbo and Scacchi, 1999).

Both populations included in the present work preserve some of the traditional characteristics linked to indigenous ancestry and local sources, such as subsistence economy (they grow vegetables and fruits to eat in the yards of their homes), fishing (fish is the main source of these proteins), and hunting (Piperata, 2007). Nowadays, fish is still the central element of the diet, usually with seven or more meals a week (Passos et al., 2008; Piperata et al., 2011; Dufour et al., 2016). Unfortunately, these food habits in Amazon can contribute to human exposure to mercury when the fish is contaminated (Passos et al., 2008; Berzas Nevado et al., 2010; Arrifano et al., 2018b). Our data revealed a significant correlation between fish intake and mercury levels, pointing to fish consumption as the origin of mercury exposure. Mercury is present in Amazon from both natural (soil) and anthropogenic sources (artisanal gold mining and dams, among others) (Wasserman et al., 2003; Berzas Nevado et al., 2010; Arrifano et al., 2018b). Once in the river, mercury undergoes biotransformation to MeHg, being incorporated in the food chain, and contaminating the fish. Therefore, it is relatively usual find mercury-intoxicated populations in Amazon (Grandjean et al., 1999; Dolbec et al., 2000; Rodrigues et al., 2007; Fillion et al., 2011b; Khoury et al., 2015).

In our study, we performed the mercury determination in hair of 757 participants (only 66 individuals abstained to donate or the collection of the sample was not possible). No correlation between mercury levels and age was found supporting the fact that age may not be an influencing factor for mercury exposure in adults. Accordingly, previous works already demonstrated no association between both factors in adults (Hoshino et al., 2015; Arrifano et al., 2018b), although it can be observed in children (Barbosa et al., 1998; Pinheiro et al., 2007; Marques et al., 2016). Moreover, it is also relatively frequent to find higher mercury levels in men than those in women, usually attributed to a higher consumption of fish (Dolbec et al., 2000; Fillion et al., 2006; Passos et al., 2007; Ashe, 2012), as we detected in our study.

Mercury levels usually found in hair varies between 0 and 2 μg/g (WHO, 2008) in non-exposed populations. In our study, median value of exposure was more than twice those levels, confirming the exposure of the population. Moreover, a substantial part of the participants (26%) showed a mercury content in hair above the limit of 10 μg/g previously recommended (Grandjean et al., 1997; Harada et al., 1999; NRC, 2000). Mercury found in hair was mainly in organic form, as MeHg. This high proportion of the organic species and the significant correlation with fish intake are in agreement with the oral exposure via contaminated fish that is found in these regions (Berzas Nevado et al., 2010; Rodriguez Martin-Doimeadios et al., 2014; Arrifano et al., 2018b). Exposure detected in Tucuruí was higher than that presently observed in Tapajós region, confirming our preliminary data with this population (Arrifano et al., 2018b).

Therefore, in this scenario of mercury exposure, the possible advantage of carrying *APOE4* would turn it on a disadvantage, with the presence of this allele implying an increased susceptibility to neurotoxicity (Godfrey et al., 2003; Wojcik et al., 2006; Ng et al., 2013, 2015; Woods et al., 2014; Snoj Tratnik et al., 2017). In our study, we found 247 individuals considered susceptible to mercury neurotoxicity, 215 with the 𝜀3/𝜀4 genotype and 32 with the 𝜀4/𝜀4 genotype. Association between the presence of *APOE4* and the worsening of mercury intoxication (including symptoms such as poorer motor performance, memory, and learning) have been well-established in human studies (see Arrifano et al., 2018a for a review). However, the events underlying this strong correlation are not totally understood. Taking into account that both factors, *APOE4* and exposure to mercury, share some similar molecular mechanisms, it was already proposed that ApoE4 would cause mainly toxicodynamic changes that could act in a synergistic way with the effects of mercury, increasing the injury and cell death (Arrifano et al., 2018a).

For the first time in Amazon, 59 individuals were identified with maximum risk showing mercury content in hair above 10 μg/g and the presence of ApoE4. Worryingly, the highest mercury content in hair found in the present work, 75.80 μg/g, was from an 𝜀4/𝜀4 individual. No difference was detected in allelic distribution of *APOE* between all participants with low and high levels of mercury (**Table 5**). Nevertheless, when the *APOE4*-carriers were compared to the *APOE2*-carriers we observed an interesting fact (**Figure 3**). When mercury exposure is below 10 μg/g, no difference in mercury levels is detected between *APOE4* and *APOE2*-carriers; however, for individuals with high levels, mercury burden was significantly higher in *APOE4* (**Figure 3**). Moreover, no association with an increased consumption in frequency or amount of fish meals in *APOE4*-carriers was detected (**Table 6**), eliminating the possibility that this bioaccumulation may be due to a higher intake of contaminated fish. Two conclusions can be reached based on our data: first, *APOE4* and *APOE2* are associated with different effects in mercury accumulation and second, the apparent pharmacokinetic influence would be of major importance with exposure above 10 μg/g.

To our knowledge, this is the first study with human populations demonstrating an association between the presence of *APOE4* and an increased accumulation of mercury. These data seems to be in agreement with the hypothesis proposed by Pendergrass and Haley (1995) suggesting that ApoE4 would show a decreased ability to bind the metal when compared to ApoE2 isoform. The organs with constitutively high content in ApoE, such as CNS, would be especially affected by this reduced ability of ApoE4 of chelating mercury. This phenomenon may facilitate the presence of the free form of the metal, allowing it to remain available and to accumulate in CNS. This would explain the increased susceptibility of *APOE4*-carriers to neurotoxicity. Although additional studies in animal models are needed to definitively establish the causal relationship, our data have already demonstrated the prerequisite for the existence of this cause-effect in humans: the presence of an association between both factors. Therefore, in addition to the possible pharmacodynamic effect, the pharmacokinetic influence of ApoE would become a key element for the worse deleterious consequences of mercury exposure.

## Conclusion

This work shows for the first time the genotypic and allelic profiles of *APOE* in Amazonian riverine populations, suggesting that the absence of 𝜀2/𝜀2 is a generalized phenomenon in Amazonian riverine populations and perhaps in the overall Amazon region. Our data support an association between *APOE4* and the Amerindian genetic background in these populations. Fifty-nine individuals were identified at maximum risk with levels of mercury above 10 μg/g and the presence of *APOE4*. This study also supports that ApoE4, in addition to a possible pharmacodynamic effect, may influence pharmacokinetically the mercury exposure causing a higher mercury bioaccumulation, which may lead to later neurodegenerative diseases with aging. All this knowledge is essential to improve prevention strategies and health policy decision-making regarding these at-risk vulnerable populations.

## Author Contributions

GA, MA-O, JS-M, BM, JA-L, JdN, RO, and MC-L discussed and conceptualized the work. GA, MA-O, JS-M, BM, LS-P, and MC-L participated in the expeditions of sample collection. RM-D, MJ-M, and SF-T quantified mercury levels. GA analyzed ApoE genotypes and alleles. MA, SS, and ÂR-d-S evaluated ancestry markers. GA, SS, and MC-L analyzed data. GA and MC-L wrote the initial draft of the manuscript and worked on subsequent revisions. GA, RM-D, MJ-M, MdO, JS-M, BM, JA-L, JdN, MA, SS, ÂR-d-S, LS-P, RO, and MC-L worked on revising the manuscript.

## Conflict of Interest Statement

The authors declare that the research was conducted in the absence of any commercial or financial relationships that could be construed as a potential conflict of interest.

## Footnotes

1 www.mercuryconvention.org

2 http://apps.who.int/gb/ebwha/pdf_files/EB134/B134_R5-en.pdf
