# Supplementary material for: Genetic Susceptibility to Neurodegeneration in Amazon: Apolipoprotein E Genotyping in Vulnerable Populations Exposed to Mercury
Source: Front Genet. 2018 Jul 27;9:285. doi: 10.3389/fgene.2018.00285 (PMC6073741; doi:10.3389/fgene.2018.00285)
Supplement: Supplementary file 1 [file Table_1.DOCX]

**Supplemental material - Table S1.** Results of rs429358 and rs7412 single-nucleotide polymorphisms for each participant of the study and APOE genotypes according to them.

| Number | Patient Code | rs429358 | rs7412 | APOE genotype |
| --- | --- | --- | --- | --- |
| 1 | 1BVT15 | T/T | C/C | E3/E3 |
| 2 | 2BVT15 | T/T | C/C | E3/E3 |
| 3 | 3BVT15 | T/T | C/C | E3/E3 |
| 4 | 4BVT15 | T/T | C/C | E3/E3 |
| 5 | 5BVT15 | T/C | C/C | E3/E4 |
| 6 | 6BVT15 | T/T | C/C | E3/E3 |
| 7 | 9BVT15 | T/T | C/C | E3/E3 |
| 8 | 10BVT15 | T/C | C/C | E3/E4 |
| 9 | 13BVT15 | T/C | C/C | E3/E4 |
| 10 | 14BVT15 | T/T | C/C | E3/E3 |
| 11 | 15BVT15 | T/T | C/C | E3/E3 |
| 12 | 16BVT15 | T/T | C/C | E3/E3 |
| 13 | 17BVT15 | T/T | C/C | E3/E3 |
| 14 | 18BVT15 | T/T | C/C | E3/E3 |
| 15 | 20BVT15 | C/C | C/C | E4/E4 |
| 16 | 22BVT15 | T/T | C/C | E3/E3 |
| 17 | 23BVT15 | T/C | C/C | E3/E4 |
| 18 | 24BVT15 | T/T | C/C | E3/E3 |
| 19 | 25BVT15 | T/T | C/C | E3/E3 |
| 20 | 26BVT15 | T/T | C/C | E3/E3 |
| 21 | 27BVT15 | T/T | C/C | E3/E3 |
| 22 | 3BRR15 | T/T | C/C | E3/E3 |
| 23 | 4BRR15 | T/T | C/C | E3/E3 |
| 24 | 6BRR15 | T/C | T/C | E2/E4 |
| 25 | 7BRR15 | T/T | T/C | E2/E3 |
| 26 | 9BRR15 | T/T | C/C | E3/E3 |
| 27 | 10BRR15 | T/T | C/C | E3/E3 |
| 28 | 11BRR15 | T/C | C/C | E3/E4 |
| 29 | 12BRR15 | T/C | C/C | E3/E4 |
| 30 | 13BRR15 | T/T | C/C | E3/E3 |
| 31 | 15BRR15 | T/T | C/C | E3/E3 |
| 32 | 16BRR15 | T/T | T/C | E2/E3 |
| 33 | 17BRR15 | T/T | C/C | E3/E3 |
| 34 | 21BRR15 | T/T | C/C | E3/E3 |
| 35 | 22BRR15 | T/T | C/C | E3/E3 |
| 36 | 23BRR15 | T/T | T/C | E2/E3 |
| 37 | 24BRR15 | T/C | C/C | E3/E4 |
| 38 | 25BRR15 | T/T | C/C | E3/E3 |
| 39 | 26BRR15 | T/T | C/C | E3/E3 |
| 40 | 28BRR15 | T/T | C/C | E3/E3 |
| 41 | 29BRR15 | T/T | C/C | E3/E3 |
| 42 | 30BRR15 | T/T | C/C | E3/E3 |
| 43 | 31BRR15 | T/T | T/C | E2/E3 |
| 44 | 32BRR15 | T/T | C/C | E3/E3 |
| 45 | 4PMT15 | T/T | C/C | E3/E3 |
| 46 | 6PMT15 | T/T | C/C | E3/E3 |
| 47 | 7PMT15 | T/T | C/C | E3/E3 |
| 48 | 9PMT15 | T/T | C/C | E3/E3 |
| 49 | 10PMT15 | T/C | C/C | E3/E4 |
| 50 | 11PMT15 | T/C | C/C | E3/E4 |
| 51 | 12PMT15 | T/C | C/C | E3/E4 |
| 52 | 13PMT15 | T/T | C/C | E3/E3 |
| 53 | 17PMT15 | T/T | C/C | E3/E3 |
| 54 | 18PMT15 | T/T | C/C | E3/E3 |
| 55 | 19PMT15 | T/C | C/C | E3/E4 |
| 56 | 21PMT15 | T/C | C/C | E3/E4 |
| 57 | 23PMT15 | T/T | C/C | E3/E3 |
| 58 | 30PMT15 | T/T | T/C | E2/E3 |
| 59 | 34PMT15 | T/C | C/C | E3/E4 |
| 60 | 2FOR15 | T/C | T/C | E2/E4 |
| 61 | 3FOR15 | T/T | C/C | E3/E3 |
| 62 | 5FOR15 | T/C | C/C | E3/E4 |
| 63 | 7FOR15 | T/T | C/C | E3/E3 |
| 64 | 11FOR15 | T/C | C/C | E3/E4 |
| 65 | 13FOR15 | T/T | C/C | E3/E3 |
| 66 | 14FOR15 | T/T | T/C | E2/E3 |
| 67 | 15FOR15 | T/T | C/C | E3/E3 |
| 68 | 16FOR15 | T/C | C/C | E3/E4 |
| 69 | 19FOR15 | T/T | C/C | E3/E3 |
| 70 | 20FOR15 | T/T | C/C | E3/E3 |
| 71 | 21FOR15 | T/T | C/C | E3/E3 |
| 72 | 22FOR15 | T/T | C/C | E3/E3 |
| 73 | 24FOR15 | T/T | C/C | E3/E3 |
| 74 | 25FOR15 | T/C | C/C | E3/E4 |
| 75 | 28FOR15 | T/T | C/C | E3/E3 |
| 76 | 29FOR15 | T/T | C/C | E3/E3 |
| 77 | 30FOR15 | T/T | C/C | E3/E3 |
| 78 | 31FOR15 | T/T | C/C | E3/E3 |
| 79 | 35FOR15 | T/T | C/C | E3/E3 |
| 80 | 36FOR15 | T/T | C/C | E3/E3 |
| 81 | 37FOR15 | T/T | C/C | E3/E3 |
| 82 | 38FOR15 | T/C | C/C | E3/E4 |
| 83 | 40FOR15 | T/C | C/C | E3/E4 |
| 84 | 41FOR15 | T/C | C/C | E3/E4 |
| 85 | 42FOR15 | C/C | C/C | E4/E4 |
| 86 | 43FOR15 | C/C | C/C | E4/E4 |
| 87 | 44FOR15 | T/T | C/C | E3/E3 |
| 88 | 45FOR15 | T/C | C/C | E3/E4 |
| 89 | 46FOR15 | T/T | C/C | E3/E3 |
| 90 | 48FOR15 | T/T | C/C | E3/E3 |
| 91 | 52FOR15 | T/T | C/C | E3/E3 |
| 92 | 53FOR15 | T/C | C/C | E3/E4 |
| 93 | 54FOR15 | T/T | C/C | E3/E3 |
| 94 | 55FOR15 | T/T | C/C | E3/E3 |
| 95 | 58FOR15 | T/T | C/C | E3/E3 |
| 96 | 60FOR15 | T/C | C/C | E3/E4 |
| 97 | 61FOR15 | T/T | C/C | E3/E3 |
| 98 | 1BSL15 | T/C | C/C | E3/E4 |
| 99 | 3BSL15 | T/T | C/C | E3/E3 |
| 100 | 4BSL15 | T/C | C/C | E3/E4 |
| 101 | 5BSL15 | T/T | T/C | E2/E3 |
| 102 | 6BSL15 | T/T | C/C | E3/E3 |
| 103 | 7BSL15 | T/C | C/C | E3/E4 |
| 104 | 8BSL15 | T/T | C/C | E3/E3 |
| 105 | 9BSL15 | C/C | C/C | E4/E4 |
| 106 | 10BSL15 | T/C | C/C | E3/E4 |
| 107 | 12BSL15 | T/T | C/C | E3/E3 |
| 108 | 13BSL15 | T/C | C/C | E3/E4 |
| 109 | 14BSL15 | T/C | C/C | E3/E4 |
| 110 | 15BSL15 | T/T | T/C | E2/E3 |
| 111 | 16BSL15 | T/C | C/C | E3/E4 |
| 112 | 18BSL15 | T/C | C/C | E3/E4 |
| 113 | 19BSL15 | T/T | C/C | E3/E3 |
| 114 | 20BSL15 | T/C | C/C | E3/E4 |
| 115 | 21BSL15 | T/C | T/C | E2/E4 |
| 116 | 22BSL15 | T/T | T/C | E2/E3 |
| 117 | 23BSL15 | T/C | C/C | E3/E4 |
| 118 | 24BSL15 | T/C | C/C | E3/E4 |
| 119 | 25BSL15 | T/C | C/C | E3/E4 |
| 120 | 26BSL15 | T/T | C/C | E3/E3 |
| 121 | 27BSL15 | T/T | C/C | E3/E3 |
| 122 | 28BSL15 | T/T | C/C | E3/E3 |
| 123 | 30BSL15 | T/T | T/C | E2/E3 |
| 124 | 31BSL15 | T/T | C/C | E3/E3 |
| 125 | 32BSL15 | T/C | T/C | E2/E4 |
| 126 | 33BSL15 | T/T | C/C | E3/E3 |
| 127 | 35BSL15 | T/T | C/C | E3/E3 |
| 128 | 38BSL15 | T/T | C/C | E3/E3 |
| 129 | 39BSL15 | T/T | C/C | E3/E3 |
| 130 | 41BSL15 | T/T | C/C | E3/E3 |
| 131 | 42BSL15 | T/T | C/C | E3/E3 |
| 132 | 43BSL15 | C/C | C/C | E4/E4 |
| 133 | 45BSL15 | T/T | C/C | E3/E3 |
| 134 | 01EBL15 | T/C | C/C | E3/E4 |
| 135 | 02EBL15 | T/T | C/C | E3/E3 |
| 136 | 03EBL15 | T/T | C/C | E3/E3 |
| 137 | 04EBL15 | T/T | C/C | E3/E3 |
| 138 | 05EBL15 | T/C | C/C | E3/E4 |
| 139 | 06EBL15 | T/T | T/C | E2/E3 |
| 140 | 07EBL15 | T/T | T/C | E2/E3 |
| 141 | 08EBL15 | T/T | C/C | E3/E3 |
| 142 | 09EBL15 | T/T | C/C | E3/E3 |
| 143 | 11EBL15 | T/T | C/C | E3/E3 |
| 144 | 12EBL15 | T/T | C/C | E3/E3 |
| 145 | 15EBL15 | T/T | C/C | E3/E3 |
| 146 | 17EBL15 | T/T | C/C | E3/E3 |
| 147 | 18EBL15 | T/C | C/C | E3/E4 |
| 148 | 19EBL15 | T/T | T/C | E2/E3 |
| 149 | 21EBL15 | T/T | T/C | E2/E3 |
| 150 | 22EBL15 | T/T | C/C | E3/E3 |
| 151 | 23EBL15 | T/C | C/C | E3/E4 |
| 152 | 24EBL15 | T/T | T/C | E2/E3 |
| 153 | 25EBL15 | T/T | C/C | E3/E3 |
| 154 | 28EBL15 | T/T | C/C | E3/E3 |
| 155 | 29EBL15 | T/T | C/C | E3/E3 |
| 156 | 32EBL15 | T/T | C/C | E3/E3 |
| 157 | 1PDB15 | T/T | C/C | E3/E3 |
| 158 | 2PDB15 | T/T | C/C | E3/E3 |
| 159 | 3PDB15 | T/C | C/C | E3/E4 |
| 160 | 4PDB15 | T/T | C/C | E3/E3 |
| 161 | 5PDB15 | T/C | C/C | E3/E4 |
| 162 | 6PDB15 | T/C | C/C | E3/E4 |
| 163 | 7PDB15 | T/T | C/C | E3/E3 |
| 164 | 8PDB15 | T/T | C/C | E3/E3 |
| 165 | 9PDB15 | T/T | C/C | E3/E3 |
| 166 | 11PDB15 | T/T | C/C | E3/E3 |
| 167 | 12PDB15 | T/T | C/C | E3/E3 |
| 168 | 13PDB15 | T/T | C/C | E3/E3 |
| 169 | 14PDB15 | T/C | C/C | E3/E4 |
| 170 | 15PDB15 | T/T | C/C | E3/E3 |
| 171 | 22PDB15 | T/T | C/C | E3/E3 |
| 172 | 23PDB15 | T/C | C/C | E3/E4 |
| 173 | 24PDB15 | T/T | C/C | E3/E3 |
| 174 | 27PDB15 | T/T | C/C | E3/E3 |
| 175 | 28PDB15 | T/T | C/C | E3/E3 |
| 176 | 29PDB15 | T/C | C/C | E3/E4 |
| 177 | 30PDB15 | T/T | C/C | E3/E3 |
| 178 | 33PDB15 | T/T | C/C | E3/E3 |
| 179 | 34PDB15 | T/C | C/C | E3/E4 |
| 180 | 35PDB15 | T/T | C/C | E3/E3 |
| 181 | 36PDB15 | T/T | C/C | E3/E3 |
| 182 | 37PDB15 | T/T | C/C | E3/E3 |
| 183 | 40PDB15 | T/T | C/C | E3/E3 |
| 184 | 41PDB15 | T/C | C/C | E3/E4 |
| 185 | 42PDB15 | T/C | C/C | E3/E4 |
| 186 | 43PDB15 | T/T | C/C | E3/E3 |
| 187 | 02EPB15 | T/T | C/C | E3/E3 |
| 188 | 03EPB15 | T/T | C/C | E3/E3 |
| 189 | 04EPB15 | T/T | C/C | E3/E3 |
| 190 | 05EPB15 | T/T | C/C | E3/E3 |
| 191 | 10EPB15 | T/T | C/C | E3/E3 |
| 192 | 12EPB15 | C/C | C/C | E4/E4 |
| 193 | 14EPB15 | T/T | C/C | E3/E3 |
| 194 | 16EPB15 | T/T | C/C | E3/E3 |
| 195 | 17EPB15 | T/T | C/C | E3/E3 |
| 196 | 18EPB15 | T/T | C/C | E3/E3 |
| 197 | 19EPB15 | T/T | C/C | E3/E3 |
| 198 | 20EPB15 | T/T | C/C | E3/E3 |
| 199 | 22EPB15 | T/T | C/C | E3/E3 |
| 200 | 1SLT16 | T/T | C/C | E3/E3 |
| 201 | 2SLT16 | T/T | T/C | E2/E3 |
| 202 | 3SLT16 | T/T | C/C | E3/E3 |
| 203 | 5SLT16 | T/T | T/C | E2/E3 |
| 204 | 6SLT16 | T/C | C/C | E3/E4 |
| 205 | 7SLT16 | T/T | C/C | E3/E3 |
| 206 | 8SLT16 | T/T | C/C | E3/E3 |
| 207 | 9SLT16 | T/T | T/C | E2/E3 |
| 208 | 10SLT16 | T/T | T/C | E2/E3 |
| 209 | 12SLT16 | T/T | C/C | E3/E3 |
| 210 | 13SLT16 | T/T | T/C | E2/E3 |
| 211 | 14SLT16 | T/C | C/C | E3/E4 |
| 212 | 15SLT16 | T/T | C/C | E3/E3 |
| 213 | 16SLT16 | T/T | C/C | E3/E3 |
| 214 | 19SLT16 | T/T | C/C | E3/E3 |
| 215 | 20SLT16 | T/C | C/C | E3/E4 |
| 216 | 21SLT16 | T/T | C/C | E3/E3 |
| 217 | 27SLT16 | T/T | C/C | E3/E3 |
| 218 | 29SLT16 | T/C | C/C | E3/E4 |
| 219 | 30SLT16 | T/T | C/C | E3/E3 |
| 220 | 33SLT16 | T/T | C/C | E3/E3 |
| 221 | 36SLT16 | C/C | C/C | E4/E4 |
| 222 | 39SLT16 | T/C | C/C | E3/E4 |
| 223 | 40SLT16 | T/T | C/C | E3/E3 |
| 224 | 41SLT16 | T/C | C/C | E3/E4 |
| 225 | 42SLT16 | T/T | C/C | E3/E3 |
| 226 | 44SLT16 | T/T | C/C | E3/E3 |
| 227 | 46SLT16 | T/C | T/C | E2/E4 |
| 228 | 49SLT16 | T/C | C/C | E3/E4 |
| 229 | 51SLT16 | T/T | C/C | E3/E3 |
| 230 | 53SLT16 | T/T | C/C | E3/E3 |
| 231 | 54SLT16 | T/C | C/C | E3/E4 |
| 232 | 55SLT16 | T/T | C/C | E3/E3 |
| 233 | 1VLR16 | T/T | C/C | E3/E3 |
| 234 | 2VLR16 | T/T | C/C | E3/E3 |
| 235 | 4VLR16 | T/T | C/C | E3/E3 |
| 236 | 8VLR16 | T/C | C/C | E3/E4 |
| 237 | 11VLR16 | T/C | C/C | E3/E4 |
| 238 | 14VLR16 | T/T | C/C | E3/E3 |
| 239 | 16VLR16 | T/T | C/C | E3/E3 |
| 240 | 18VLR16 | T/T | C/C | E3/E3 |
| 241 | 19VLR16 | T/C | C/C | E3/E4 |
| 242 | 22VLR16 | T/C | C/C | E3/E4 |
| 243 | 23VLR16 | T/C | C/C | E3/E4 |
| 244 | 3NVC16 | T/T | C/C | E3/E3 |
| 245 | 5NVC16 | T/T | C/C | E3/E3 |
| 246 | 7NVC16 | T/T | C/C | E3/E3 |
| 247 | 1BRR16 | T/T | C/C | E3/E3 |
| 248 | 3BRR16 | T/T | C/C | E3/E3 |
| 249 | 4BRR16 | T/T | C/C | E3/E3 |
| 250 | 5BRR16 | T/C | C/C | E3/E4 |
| 251 | 10BRR16 | T/C | C/C | E3/E4 |
| 252 | 11BRR16 | C/C | C/C | E4/E4 |
| 253 | 12BRR16 | T/T | T/C | E2/E3 |
| 254 | 13BRR16 | T/T | C/C | E3/E3 |
| 255 | 14BRR16 | T/T | C/C | E3/E3 |
| 256 | 16BRR16 | T/T | C/C | E3/E3 |
| 257 | 17BRR16 | T/T | C/C | E3/E3 |
| 258 | 18BRR16 | C/C | C/C | E4/E4 |
| 259 | 19BRR16 | T/C | C/C | E3/E4 |
| 260 | 20BRR16 | T/T | C/C | E3/E3 |
| 261 | 22BRR16 | T/T | C/C | E3/E3 |
| 262 | 23BRR16 | T/T | C/C | E3/E3 |
| 263 | 24BRR16 | T/C | C/C | E3/E4 |
| 264 | 26BRR16 | T/C | C/C | E3/E4 |
| 265 | 27BRR16 | T/T | C/C | E3/E3 |
| 266 | 29BRR16 | C/C | C/C | E4/E4 |
| 267 | 30BRR16 | T/C | C/C | E3/E4 |
| 268 | 31BRR16 | T/T | C/C | E3/E3 |
| 269 | 35BRR16 | T/C | C/C | E3/E4 |
| 270 | 36BRR16 | T/T | C/C | E3/E3 |
| 271 | 37BRR16 | T/T | T/C | E2/E3 |
| 272 | 38BRR16 | T/T | C/C | E3/E3 |
| 273 | 41BRR16 | T/T | T/C | E2/E3 |
| 274 | 48BRR16 | T/C | C/C | E3/E4 |
| 275 | 52BRR16 | T/T | T/C | E2/E3 |
| 276 | 53BRR16 | T/C | C/C | E3/E4 |
| 277 | 54BRR16 | T/T | C/C | E3/E3 |
| 278 | 57BRR16 | T/T | C/C | E3/E3 |
| 279 | 58BRR16 | T/T | T/C | E2/E3 |
| 280 | 59BRR16 | T/T | C/C | E3/E3 |
| 281 | 60BRR16 | T/T | C/C | E3/E3 |
| 282 | 65BRR16 | T/T | C/C | E3/E3 |
| 283 | 66BRR16 | T/T | C/C | E3/E3 |
| 284 | 70BRR16 | T/T | C/C | E3/E3 |
| 285 | 71BRR16 | T/T | C/C | E3/E3 |
| 286 | 72BRR16 | T/T | C/C | E3/E3 |
| 287 | 73BRR16 | T/T | C/C | E3/E3 |
| 288 | 75BRR16 | T/C | T/C | E2/E4 |
| 289 | 76BRR16 | T/T | C/C | E3/E3 |
| 290 | 77BRR16 | T/T | T/C | E2/E3 |
| 291 | 79BRR16 | T/T | C/C | E3/E3 |
| 292 | 80BRR16 | T/C | C/C | E3/E4 |
| 293 | 84BRR16 | T/T | C/C | E3/E3 |
| 294 | 90BRR16 | T/C | C/C | E3/E4 |
| 295 | 95BRR16 | T/C | C/C | E3/E4 |
| 296 | 7BVT15 | T/C | C/C | E3/E4 |
| 297 | 8BVT15 | T/T | T/C | E2/E3 |
| 298 | 12BVT15 | T/T | C/C | E3/E3 |
| 299 | 19BVT15 | T/T | C/C | E3/E3 |
| 300 | 21BVT15 | T/T | C/C | E3/E3 |
| 301 | 28BVT15 | T/T | C/C | E3/E3 |
| 302 | 29BVT15 | T/T | C/C | E3/E3 |
| 303 | 30BVT15 | T/C | C/C | E3/E4 |
| 304 | 1BRR15 | T/T | C/C | E3/E3 |
| 305 | 2BRR15 | T/C | C/C | E3/E4 |
| 306 | 5BRR15 | T/T | C/C | E3/E3 |
| 307 | 8BRR15 | T/T | C/C | E3/E3 |
| 308 | 14BRR15 | T/T | C/C | E3/E3 |
| 309 | 18BRR15 | T/T | C/C | E3/E3 |
| 310 | 19BRR15 | T/T | C/C | E3/E3 |
| 311 | 27BRR15 | T/C | C/C | E3/E4 |
| 312 | 2PMT15 | T/C | C/C | E3/E4 |
| 313 | 3PMT15 | T/T | C/C | E3/E3 |
| 314 | 5PMT15 | T/T | T/C | E2/E3 |
| 315 | 8PMT15 | T/T | C/C | E3/E3 |
| 316 | 14PMT15 | T/T | C/C | E3/E3 |
| 317 | 15PMT15 | T/C | T/C | E2/E4 |
| 318 | 16PMT15 | T/T | C/C | E3/E3 |
| 319 | 20PMT15 | T/C | C/C | E3/E4 |
| 320 | 22PMT15 | T/T | C/C | E3/E3 |
| 321 | 27PMT15 | T/T | C/C | E3/E3 |
| 322 | 28PMT15 | T/T | C/C | E3/E3 |
| 323 | 31PMT15 | T/T | C/C | E3/E3 |
| 324 | 32PMT15 | T/T | C/C | E3/E3 |
| 325 | 33PMT15 | T/C | C/C | E3/E4 |
| 326 | 1FOR15 | T/C | C/C | E3/E4 |
| 327 | 4FOR15 | T/T | C/C | E3/E3 |
| 328 | 6FOR15 | T/C | C/C | E3/E4 |
| 329 | 8FOR15 | T/T | C/C | E3/E3 |
| 330 | 9FOR15 | T/T | C/C | E3/E3 |
| 331 | 10FOR15 | T/T | C/C | E3/E3 |
| 332 | 17FOR15 | T/T | C/C | E3/E3 |
| 333 | 18FOR15 | T/T | C/C | E3/E3 |
| 334 | 23FOR15 | T/T | C/C | E3/E3 |
| 335 | 26FOR15 | T/T | C/C | E3/E3 |
| 336 | 27FOR15 | T/T | C/C | E3/E3 |
| 337 | 32FOR15 | T/C | C/C | E3/E4 |
| 338 | 33FOR15 | T/T | C/C | E3/E3 |
| 339 | 39FOR15 | C/C | C/C | E4/E4 |
| 340 | 47FOR15 | T/T | T/C | E2/E3 |
| 341 | 49FOR15 | T/T | C/C | E3/E3 |
| 342 | 50FOR15 | T/T | C/C | E3/E3 |
| 343 | 51FOR15 | T/T | C/C | E3/E3 |
| 344 | 56FOR15 | T/T | C/C | E3/E3 |
| 345 | 57FOR15 | T/C | C/C | E3/E4 |
| 346 | 59FOR15 | T/T | C/C | E3/E3 |
| 347 | 11BSL15 | T/C | C/C | E3/E4 |
| 348 | 17BSL15 | T/T | C/C | E3/E3 |
| 349 | 29BSL15 | T/T | C/C | E3/E3 |
| 350 | 34BSL15 | T/C | C/C | E3/E4 |
| 351 | 36BSL15 | T/C | C/C | E3/E4 |
| 352 | 37BSL15 | T/C | C/C | E3/E4 |
| 353 | 40BSL15 | T/C | C/C | E3/E4 |
| 354 | 44BSL15 | T/T | C/C | E3/E3 |
| 355 | 10EBL15 | T/T | T/C | E2/E3 |
| 356 | 16EBL15 | T/T | C/C | E3/E3 |
| 357 | 26EBL15 | T/C | T/C | E2/E4 |
| 358 | 27EBL15 | T/C | C/C | E3/E4 |
| 359 | 30EBL15 | T/C | C/C | E3/E4 |
| 360 | 31EBL15 | T/T | C/C | E3/E3 |
| 361 | 33EBL15 | T/C | C/C | E3/E4 |
| 362 | 10PDB15 | T/T | C/C | E3/E3 |
| 363 | 18PDB15 | T/T | C/C | E3/E3 |
| 364 | 19PDB15 | T/C | C/C | E3/E4 |
| 365 | 20PDB15 | T/T | T/C | E2/E3 |
| 366 | 21PDB15 | T/T | C/C | E3/E3 |
| 367 | 25PDB15 | T/T | C/C | E3/E3 |
| 368 | 26PDB15 | T/T | C/C | E3/E3 |
| 369 | 31PDB15 | T/T | C/C | E3/E3 |
| 370 | 32PDB15 | T/T | C/C | E3/E3 |
| 371 | 38PDB15 | T/T | C/C | E3/E3 |
| 372 | 39PDB15 | T/T | C/C | E3/E3 |
| 373 | 44PDB15 | T/C | C/C | E3/E4 |
| 374 | 06EPB15 | T/T | C/C | E3/E3 |
| 375 | 07EPB15 | T/C | C/C | E3/E4 |
| 376 | 09EPB15 | T/T | C/C | E3/E3 |
| 377 | 13EPB15 | T/T | C/C | E3/E3 |
| 378 | 15EPB15 | T/T | C/C | E3/E3 |
| 379 | 21EPB15 | T/T | C/C | E3/E3 |
| 380 | 4SLT16 | T/C | C/C | E3/E4 |
| 381 | 11SLT16 | T/C | C/C | E3/E4 |
| 382 | 17SLT16 | T/T | T/C | E2/E3 |
| 383 | 18SLT16 | T/T | C/C | E3/E3 |
| 384 | 22SLT16 | T/T | C/C | E3/E3 |
| 385 | 23SLT16 | T/T | C/C | E3/E3 |
| 386 | 25SLT16 | T/T | C/C | E3/E3 |
| 387 | 32SLT16 | T/T | C/C | E3/E3 |
| 388 | 34SLT16 | T/C | C/C | E3/E4 |
| 389 | 35SLT16 | T/T | C/C | E3/E3 |
| 390 | 37SLT16 | T/C | C/C | E3/E4 |
| 391 | 38SLT16 | T/T | C/C | E3/E3 |
| 392 | 43SLT16 | T/T | C/C | E3/E3 |
| 393 | 45SLT16 | T/C | C/C | E3/E4 |
| 394 | 47SLT16 | T/C | C/C | E3/E4 |
| 395 | 48SLT16 | T/T | C/C | E3/E3 |
| 396 | 50SLT16 | T/C | C/C | E3/E4 |
| 397 | 52SLT16 | T/T | C/C | E3/E3 |
| 398 | 3VLR16 | T/T | C/C | E3/E3 |
| 399 | 5VLR16 | T/C | C/C | E3/E4 |
| 400 | 6VLR16 | T/C | C/C | E3/E4 |
| 401 | 7VLR16 | T/C | C/C | E3/E4 |
| 402 | 9VLR16 | T/T | C/C | E3/E3 |
| 403 | 10VLR16 | T/C | C/C | E3/E4 |
| 404 | 12VLR16 | T/T | C/C | E3/E3 |
| 405 | 13VLR16 | T/C | C/C | E3/E4 |
| 406 | 15VLR16 | T/T | C/C | E3/E3 |
| 407 | 17VLR16 | T/C | C/C | E3/E4 |
| 408 | 20VLR16 | T/T | C/C | E3/E3 |
| 409 | 21VLR16 | T/T | C/C | E3/E3 |
| 410 | 24VLR16 | T/T | C/C | E3/E3 |
| 411 | 1NVC16 | T/T | T/C | E2/E3 |
| 412 | 2NVC16 | T/T | C/C | E3/E3 |
| 413 | 4NVC16 | T/C | C/C | E3/E4 |
| 414 | 6NVC16 | T/T | C/C | E3/E3 |
| 415 | 8NVC16 | T/T | T/C | E2/E3 |
| 416 | 6BRR16 | T/C | C/C | E3/E4 |
| 417 | 8BRR16 | T/T | C/C | E3/E3 |
| 418 | 9BRR16 | T/C | C/C | E3/E4 |
| 419 | 15BRR16 | T/T | C/C | E3/E3 |
| 420 | 32BRR16 | T/T | C/C | E3/E3 |
| 421 | 39BRR16 | T/C | C/C | E3/E4 |
| 422 | 40BRR16 | T/T | C/C | E3/E3 |
| 423 | 42BRR16 | T/T | C/C | E3/E3 |
| 424 | 43BRR16 | T/C | C/C | E3/E4 |
| 425 | 44BRR16 | T/T | C/C | E3/E3 |
| 426 | 45BRR16 | T/T | T/C | E2/E3 |
| 427 | 46BRR16 | T/T | C/C | E3/E3 |
| 428 | 47BRR16 | T/T | C/C | E3/E3 |
| 429 | 49BRR16 | T/C | C/C | E3/E4 |
| 430 | 51BRR16 | T/T | C/C | E3/E3 |
| 431 | 61BRR16 | T/T | C/C | E3/E3 |
| 432 | 62BRR16 | T/T | C/C | E3/E3 |
| 433 | 67BRR16 | T/T | C/C | E3/E3 |
| 434 | 68BRR16 | T/T | C/C | E3/E3 |
| 435 | 69BRR16 | T/T | C/C | E3/E3 |
| 436 | 78BRR16 | T/T | T/C | E2/E3 |
| 437 | 81BRR16 | T/T | C/C | E3/E3 |
| 438 | 82BRR16 | T/T | C/C | E3/E3 |
| 439 | 83BRR16 | T/T | C/C | E3/E3 |
| 440 | 85BRR16 | T/C | C/C | E3/E4 |
| 441 | 89BRR16 | T/C | T/C | E2/E4 |
| 442 | 91BRR16 | T/T | T/C | E2/E3 |
| 443 | 92BRR16 | T/C | C/C | E3/E4 |
| 444 | 93BRR16 | T/T | C/C | E3/E3 |
| 445 | 96BRR16 | T/T | C/C | E3/E3 |
| 446 | 97BRR16 | T/T | T/C | E2/E3 |
| 447 | 5TVC15 | T/T | C/C | E3/E3 |
| 448 | 6TVC15 | T/T | C/C | E3/E3 |
| 449 | 7TVC15 | T/C | C/C | E3/E4 |
| 450 | 8TVC15 | T/T | C/C | E3/E3 |
| 451 | 10TVC15 | T/C | C/C | E3/E4 |
| 452 | 12TVC15 | T/T | C/C | E3/E3 |
| 453 | 13TVC15 | T/T | C/C | E3/E3 |
| 454 | 14TVC15 | T/T | C/C | E3/E3 |
| 455 | 15TVC15 | T/C | C/C | E3/E4 |
| 456 | 16TVC15 | T/T | C/C | E3/E3 |
| 457 | 21TVOV15 | T/T | T/C | E2/E3 |
| 458 | 23TVOV15 | T/C | C/C | E3/E4 |
| 459 | 25TVOV15 | T/T | C/C | E3/E3 |
| 460 | 26TVOV15 | T/T | C/C | E3/E3 |
| 461 | 30TVOV15 | T/T | C/C | E3/E3 |
| 462 | 31TVOV15 | T/T | C/C | E3/E3 |
| 463 | 32TVOV15 | T/T | C/C | E3/E3 |
| 464 | 34TVOV15 | T/C | C/C | E3/E4 |
| 465 | 37TVOV15 | T/T | C/C | E3/E3 |
| 466 | 01TUC16 | T/T | C/C | E3/E3 |
| 467 | 02TUC16 | T/C | C/C | E3/E4 |
| 468 | 05TUC16 | T/T | C/C | E3/E3 |
| 469 | 07TUC16 | T/T | C/C | E3/E3 |
| 470 | 08TUC16 | T/C | C/C | E3/E4 |
| 471 | 09TUC16 | T/T | T/C | E2/E3 |
| 472 | 11TUC16 | T/T | T/C | E2/E3 |
| 473 | 13TUC16 | T/T | C/C | E3/E3 |
| 474 | 14TUC16 | T/T | C/C | E3/E3 |
| 475 | 20TUC16 | T/T | C/C | E3/E3 |
| 476 | 21TUC16 | T/T | C/C | E3/E3 |
| 477 | 23TUC16 | T/C | C/C | E3/E4 |
| 478 | 26TUC16 | T/C | C/C | E3/E4 |
| 479 | 28TUC16 | T/T | C/C | E3/E3 |
| 480 | 29TUC16 | T/T | C/C | E3/E3 |
| 481 | 31TUC16 | T/T | C/C | E3/E3 |
| 482 | 32TUC16 | T/T | C/C | E3/E3 |
| 483 | 33TUC16 | C/C | C/C | E4/E4 |
| 484 | 35TUC16 | T/C | C/C | E3/E4 |
| 485 | 41TUC16 | C/C | C/C | E4/E4 |
| 486 | 42TUC16 | T/C | C/C | E3/E4 |
| 487 | 43TUC16 | T/T | C/C | E3/E3 |
| 488 | 49TUC16 | T/T | C/C | E3/E3 |
| 489 | 50TUC16 | T/T | C/C | E3/E3 |
| 490 | 52TUC16 | T/T | C/C | E3/E3 |
| 491 | 53TUC16 | T/T | C/C | E3/E3 |
| 492 | 54TUC16 | C/C | C/C | E4/E4 |
| 493 | 55TUC16 | C/C | C/C | E4/E4 |
| 494 | 56TUC16 | T/C | C/C | E3/E4 |
| 495 | 57TUC16 | T/T | T/C | E2/E3 |
| 496 | 59TUC16 | T/T | C/C | E3/E3 |
| 497 | 61TUC16 | T/C | C/C | E3/E4 |
| 498 | 63TUC16 | C/C | C/C | E4/E4 |
| 499 | 66TUC16 | T/T | C/C | E3/E3 |
| 500 | 67TUC16 | C/C | C/C | E4/E4 |
| 501 | 69TUC16 | T/T | C/C | E3/E3 |
| 502 | 70TUC16 | T/C | C/C | E3/E4 |
| 503 | 72TUC16 | T/T | C/C | E3/E3 |
| 504 | 73TUC16 | T/T | C/C | E3/E3 |
| 505 | 75TUC16 | T/T | C/C | E3/E3 |
| 506 | 77TUC16 | C/C | C/C | E4/E4 |
| 507 | 78TUC16 | T/T | C/C | E3/E3 |
| 508 | 81TUC16 | T/C | T/C | E2/E4 |
| 509 | 83TUC16 | T/T | C/C | E3/E3 |
| 510 | 89TUC16 | T/C | C/C | E3/E4 |
| 511 | 92TUC16 | T/T | C/C | E3/E3 |
| 512 | 95TUC16 | T/C | C/C | E3/E4 |
| 513 | 96TUC16 | T/C | C/C | E3/E4 |
| 514 | 97TUC16 | T/T | C/C | E3/E3 |
| 515 | 99TUC16 | T/C | C/C | E3/E4 |
| 516 | 101TUC16 | T/T | C/C | E3/E3 |
| 517 | 103TUC16 | T/C | C/C | E3/E4 |
| 518 | 104TUC16 | T/T | C/C | E3/E3 |
| 519 | 105TUC16 | T/C | C/C | E3/E4 |
| 520 | 106TUC16 | C/C | C/C | E4/E4 |
| 521 | 109TUC16 | T/C | C/C | E3/E4 |
| 522 | 110TUC16 | T/T | C/C | E3/E3 |
| 523 | 111TUC16 | T/T | C/C | E3/E3 |
| 524 | 112TUC16 | T/C | C/C | E3/E4 |
| 525 | 113TUC16 | T/T | C/C | E3/E3 |
| 526 | 114TUC16 | T/T | C/C | E3/E3 |
| 527 | 115TUC16 | T/T | C/C | E3/E3 |
| 528 | 117TUC16 | T/T | C/C | E3/E3 |
| 529 | 118TUC16 | T/T | C/C | E3/E3 |
| 530 | 120TUC16 | T/T | C/C | E3/E3 |
| 531 | 122TUC16 | T/T | C/C | E3/E3 |
| 532 | 123TUC16 | T/T | C/C | E3/E3 |
| 533 | 125TUC16 | T/T | T/C | E2/E3 |
| 534 | 126TUC16 | T/C | C/C | E3/E4 |
| 535 | 127TUC16 | T/C | C/C | E3/E4 |
| 536 | 131TUC16 | T/T | C/C | E3/E3 |
| 537 | 132TUC16 | T/T | C/C | E3/E3 |
| 538 | 133TUC16 | T/T | C/C | E3/E3 |
| 539 | 135TUC16 | T/T | C/C | E3/E3 |
| 540 | 136TUC16 | T/T | C/C | E3/E3 |
| 541 | 139TUC16 | T/T | C/C | E3/E3 |
| 542 | 140TUC16 | T/C | C/C | E3/E4 |
| 543 | 141TUC16 | T/C | T/C | E2/E4 |
| 544 | 145TUC16 | T/T | C/C | E3/E3 |
| 545 | 149TUC16 | T/T | T/C | E2/E3 |
| 546 | 154TUC16 | T/T | T/C | E2/E3 |
| 547 | 156TUC16 | T/T | C/C | E3/E3 |
| 548 | 157TUC16 | T/T | C/C | E3/E3 |
| 549 | 158TUC16 | T/T | C/C | E3/E3 |
| 550 | 159TUC16 | T/T | C/C | E3/E3 |
| 551 | 161TUC16 | T/T | C/C | E3/E3 |
| 552 | 163TUC16 | T/T | C/C | E3/E3 |
| 553 | 169TUC16 | T/T | C/C | E3/E3 |
| 554 | 171TUC16 | T/T | C/C | E3/E3 |
| 555 | 175TUC16 | T/T | T/C | E2/E3 |
| 556 | 176TUC16 | T/T | C/C | E3/E3 |
| 557 | 177TUC16 | T/T | C/C | E3/E3 |
| 558 | 178TUC16 | T/T | C/C | E3/E3 |
| 559 | 179TUC16 | T/T | C/C | E3/E3 |
| 560 | 180TUC16 | T/C | C/C | E3/E4 |
| 561 | 181TUC16 | T/T | C/C | E3/E3 |
| 562 | 01TUC17 | T/T | C/C | E3/E3 |
| 563 | 03TUC17 | T/C | C/C | E3/E4 |
| 564 | 07TUC17 | T/C | C/C | E3/E4 |
| 565 | 09TUC17 | T/T | C/C | E3/E3 |
| 566 | 11TUC17 | T/T | C/C | E3/E3 |
| 567 | 12TUC17 | T/T | T/C | E2/E3 |
| 568 | 13TUC17 | T/T | C/C | E3/E3 |
| 569 | 15TUC17 | T/C | C/C | E3/E4 |
| 570 | 16TUC17 | T/T | C/C | E3/E3 |
| 571 | 18TUC17 | T/T | C/C | E3/E3 |
| 572 | 19TUC17 | T/T | C/C | E3/E3 |
| 573 | 20TUC17 | T/T | C/C | E3/E3 |
| 574 | 21TUC17 | T/T | T/C | E2/E3 |
| 575 | 22TUC17 | T/C | C/C | E3/E4 |
| 576 | 23TUC17 | T/C | C/C | E3/E4 |
| 577 | 24TUC17 | T/T | C/C | E3/E3 |
| 578 | 27TUC17 | T/T | C/C | E3/E3 |
| 579 | 29TUC17 | T/T | C/C | E3/E3 |
| 580 | 30TUC17 | T/T | C/C | E3/E3 |
| 581 | 33TUC17 | T/T | C/C | E3/E3 |
| 582 | 35TUC17 | T/C | C/C | E3/E4 |
| 583 | 36TUC17 | T/T | C/C | E3/E3 |
| 584 | 38TUC17 | T/T | T/C | E2/E3 |
| 585 | 40TUC17 | T/C | T/C | E2/E4 |
| 586 | 41TUC17 | T/T | C/C | E3/E3 |
| 587 | 42TUC17 | T/T | C/C | E3/E3 |
| 588 | 43TUC17 | T/C | C/C | E3/E4 |
| 589 | 46TUC17 | C/C | C/C | E4/E4 |
| 590 | 49TUC17 | T/T | C/C | E3/E3 |
| 591 | 50TUC17 | T/C | C/C | E3/E4 |
| 592 | 52TUC17 | T/C | C/C | E3/E4 |
| 593 | 53TUC17 | T/T | C/C | E3/E3 |
| 594 | 54TUC17 | T/T | C/C | E3/E3 |
| 595 | 56TUC17 | T/T | C/C | E3/E3 |
| 596 | 58TUC17 | C/C | C/C | E4/E4 |
| 597 | 65TUC17 | T/T | C/C | E3/E3 |
| 598 | 66TUC17 | T/T | C/C | E3/E3 |
| 599 | 68TUC17 | T/C | T/C | E2/E4 |
| 600 | 71TUC17 | T/T | C/C | E3/E3 |
| 601 | 72TUC17 | T/T | C/C | E3/E3 |
| 602 | 74TUC17 | T/T | C/C | E3/E3 |
| 603 | 76TUC17 | T/C | C/C | E3/E4 |
| 604 | 77TUC17 | C/C | C/C | E4/E4 |
| 605 | 79TUC17 | T/C | C/C | E3/E4 |
| 606 | 80TUC17 | T/T | C/C | E3/E3 |
| 607 | 81TUC17 | T/C | C/C | E3/E4 |
| 608 | 84TUC17 | T/C | C/C | E3/E4 |
| 609 | 86TUC17 | T/T | C/C | E3/E3 |
| 610 | 91TUC17 | T/C | C/C | E3/E4 |
| 611 | 100TUC17 | T/T | C/C | E3/E3 |
| 612 | 102TUC17 | T/C | C/C | E3/E4 |
| 613 | 103TUC17 | T/T | C/C | E3/E3 |
| 614 | 105TUC17 | T/T | C/C | E3/E3 |
| 615 | 106TUC17 | T/C | C/C | E3/E4 |
| 616 | 107TUC17 | C/C | C/C | E4/E4 |
| 617 | 109TUC17 | T/C | C/C | E3/E4 |
| 618 | 112TUC17 | T/C | C/C | E3/E4 |
| 619 | 115TUC17 | T/T | C/C | E3/E3 |
| 620 | 116TUC17 | T/T | C/C | E3/E3 |
| 621 | 118TUC17 | T/T | T/C | E2/E3 |
| 622 | 119TUC17 | T/C | C/C | E3/E4 |
| 623 | 120TUC17 | T/C | C/C | E3/E4 |
| 624 | 121TUC17 | T/C | C/C | E3/E4 |
| 625 | 122TUC17 | T/T | C/C | E3/E3 |
| 626 | 125TUC17 | T/T | C/C | E3/E3 |
| 627 | 126TUC17 | T/C | C/C | E3/E4 |
| 628 | 128TUC17 | T/C | C/C | E3/E4 |
| 629 | 129TUC17 | T/C | C/C | E3/E4 |
| 630 | 130TUC17 | C/C | C/C | E4/E4 |
| 631 | 131TUC17 | T/T | T/C | E2/E3 |
| 632 | 133TUC17 | T/C | C/C | E3/E4 |
| 633 | 134TUC17 | C/C | C/C | E4/E4 |
| 634 | 137TUC17 | T/T | C/C | E3/E3 |
| 635 | 139TUC17 | T/T | C/C | E3/E3 |
| 636 | 140TUC17 | T/T | C/C | E3/E3 |
| 637 | 141TUC17 | T/C | C/C | E3/E4 |
| 638 | 142TUC17 | T/T | C/C | E3/E3 |
| 639 | 145TUC17 | T/T | C/C | E3/E3 |
| 640 | 146TUC17 | T/T | C/C | E3/E3 |
| 641 | 151TUC17 | T/C | C/C | E3/E4 |
| 642 | 152TUC17 | T/T | C/C | E3/E3 |
| 643 | 153TUC17 | T/T | C/C | E3/E3 |
| 644 | 155TUC17 | T/C | C/C | E3/E4 |
| 645 | 156TUC17 | T/T | T/C | E2/E3 |
| 646 | 158TUC17 | T/T | C/C | E3/E3 |
| 647 | 160TUC17 | T/T | C/C | E3/E3 |
| 648 | 161TUC17 | T/T | T/C | E2/E3 |
| 649 | 162TUC17 | T/C | C/C | E3/E4 |
| 650 | 163TUC17 | T/T | C/C | E3/E3 |
| 651 | 1TVC15 | T/T | C/C | E3/E3 |
| 652 | 2TVC15 | T/T | T/C | E2/E3 |
| 653 | 3TVC15 | T/T | C/C | E3/E3 |
| 654 | 4TVC15 | T/C | C/C | E3/E4 |
| 655 | 9TVC15 | T/T | C/C | E3/E3 |
| 656 | 11TVC15 | T/C | C/C | E3/E4 |
| 657 | 17TVC15 | T/C | C/C | E3/E4 |
| 658 | 18TVOV15 | T/T | C/C | E3/E3 |
| 659 | 19TVOV15 | T/C | C/C | E3/E4 |
| 660 | 20TVOV15 | C/C | C/C | E4/E4 |
| 661 | 22TVOV15 | T/C | C/C | E3/E4 |
| 662 | 24TVOV15 | T/T | C/C | E3/E3 |
| 663 | 27TVOV15 | T/C | C/C | E3/E4 |
| 664 | 28TVOV15 | T/C | C/C | E3/E4 |
| 665 | 29TVOV15 | T/T | C/C | E3/E3 |
| 666 | 33TVOV15 | T/C | C/C | E3/E4 |
| 667 | 35TVOV15 | T/T | C/C | E3/E3 |
| 668 | 36TVOV15 | T/T | C/C | E3/E3 |
| 669 | 38TVOV15 | T/T | C/C | E3/E3 |
| 670 | 03TUC16 | T/C | C/C | E3/E4 |
| 671 | 04TUC16 | T/T | C/C | E3/E3 |
| 672 | 06TUC16 | T/T | C/C | E3/E3 |
| 673 | 10TUC16 | T/T | C/C | E3/E3 |
| 674 | 12TUC16 | T/T | C/C | E3/E3 |
| 675 | 15TUC16 | T/C | C/C | E3/E4 |
| 676 | 16TUC16 | T/T | C/C | E3/E3 |
| 677 | 17TUC16 | T/T | C/C | E3/E3 |
| 678 | 18TUC16 | T/T | C/C | E3/E3 |
| 679 | 19TUC16 | T/C | C/C | E3/E4 |
| 680 | 22TUC16 | C/C | C/C | E4/E4 |
| 681 | 25TUC16 | T/T | C/C | E3/E3 |
| 682 | 27TUC16 | T/T | C/C | E3/E3 |
| 683 | 34TUC16 | T/C | C/C | E3/E4 |
| 684 | 36TUC16 | T/T | C/C | E3/E3 |
| 685 | 37TUC16 | T/T | C/C | E3/E3 |
| 686 | 38TUC16 | T/C | C/C | E3/E4 |
| 687 | 39TUC16 | T/T | C/C | E3/E3 |
| 688 | 40TUC16 | T/C | C/C | E3/E4 |
| 689 | 44TUC16 | T/T | C/C | E3/E3 |
| 690 | 45TUC16 | T/C | C/C | E3/E4 |
| 691 | 46TUC16 | T/C | C/C | E3/E4 |
| 692 | 51TUC16 | T/T | T/C | E2/E3 |
| 693 | 58TUC16 | T/T | C/C | E3/E3 |
| 694 | 60TUC16 | T/C | C/C | E3/E4 |
| 695 | 62TUC16 | T/T | C/C | E3/E3 |
| 696 | 65TUC16 | T/T | T/C | E2/E3 |
| 697 | 68TUC16 | T/T | C/C | E3/E3 |
| 698 | 71TUC16 | T/T | C/C | E3/E3 |
| 699 | 74TUC16 | T/T | C/C | E3/E3 |
| 700 | 76TUC16 | T/C | C/C | E3/E4 |
| 701 | 79TUC16 | C/C | C/C | E4/E4 |
| 702 | 80TUC16 | T/T | C/C | E3/E3 |
| 703 | 82TUC16 | T/T | C/C | E3/E3 |
| 704 | 84TUC16 | T/T | C/C | E3/E3 |
| 705 | 85TUC16 | T/T | C/C | E3/E3 |
| 706 | 86TUC16 | T/T | C/C | E3/E3 |
| 707 | 91TUC16 | T/C | C/C | E3/E4 |
| 708 | 98TUC16 | T/C | C/C | E3/E4 |
| 709 | 102TUC16 | T/C | C/C | E3/E4 |
| 710 | 107TUC16 | T/T | C/C | E3/E3 |
| 711 | 108TUC16 | T/C | C/C | E3/E4 |
| 712 | 116TUC16 | T/C | C/C | E3/E4 |
| 713 | 119TUC16 | T/C | C/C | E3/E4 |
| 714 | 121TUC16 | C/C | C/C | E4/E4 |
| 715 | 124TUC16 | T/C | C/C | E3/E4 |
| 716 | 128TUC16 | T/C | C/C | E3/E4 |
| 717 | 129TUC16 | T/T | C/C | E3/E3 |
| 718 | 130TUC16 | T/T | C/C | E3/E3 |
| 719 | 134TUC16 | T/T | C/C | E3/E3 |
| 720 | 138TUC16 | T/T | T/C | E2/E3 |
| 721 | 143TUC16 | T/T | C/C | E3/E3 |
| 722 | 144TUC16 | T/C | C/C | E3/E4 |
| 723 | 146TUC16 | T/T | C/C | E3/E3 |
| 724 | 148TUC16 | T/T | C/C | E3/E3 |
| 725 | 150TUC16 | T/T | T/C | E2/E3 |
| 726 | 151TUC16 | T/C | C/C | E3/E4 |
| 727 | 152TUC16 | T/C | C/C | E3/E4 |
| 728 | 153TUC16 | T/T | C/C | E3/E3 |
| 729 | 155TUC16 | T/C | C/C | E3/E4 |
| 730 | 164TUC16 | T/T | C/C | E3/E3 |
| 731 | 165TUC16 | T/T | C/C | E3/E3 |
| 732 | 166TUC16 | T/T | C/C | E3/E3 |
| 733 | 167TUC16 | T/T | C/C | E3/E3 |
| 734 | 168TUC16 | T/T | C/C | E3/E3 |
| 735 | 172TUC16 | C/C | C/C | E4/E4 |
| 736 | 173TUC16 | T/T | C/C | E3/E3 |
| 737 | 174TUC16 | T/T | C/C | E3/E3 |
| 738 | 182TUC16 | T/C | C/C | E3/E4 |
| 739 | 183TUC16 | T/T | C/C | E3/E3 |
| 740 | 02TUC17 | T/T | C/C | E3/E3 |
| 741 | 04TUC17 | C/C | C/C | E4/E4 |
| 742 | 05TUC17 | T/T | C/C | E3/E3 |
| 743 | 08TUC17 | T/T | T/C | E2/E3 |
| 744 | 10TUC17 | T/T | C/C | E3/E3 |
| 745 | 14TUC17 | T/C | C/C | E3/E4 |
| 746 | 17TUC17 | T/T | C/C | E3/E3 |
| 747 | 25TUC17 | T/C | C/C | E3/E4 |
| 748 | 26TUC17 | T/T | C/C | E3/E3 |
| 749 | 28TUC17 | T/C | C/C | E3/E4 |
| 750 | 31TUC17 | T/T | T/C | E2/E3 |
| 751 | 32TUC17 | T/T | C/C | E3/E3 |
| 752 | 34TUC17 | T/C | C/C | E3/E4 |
| 753 | 44TUC17 | T/T | C/C | E3/E3 |
| 754 | 45TUC17 | T/T | C/C | E3/E3 |
| 755 | 51TUC17 | T/T | T/C | E2/E3 |
| 756 | 55TUC17 | T/T | C/C | E3/E3 |
| 757 | 64TUC17 | T/T | C/C | E3/E3 |
| 758 | 67TUC17 | T/C | C/C | E3/E4 |
| 759 | 69TUC17 | T/T | C/C | E3/E3 |
| 760 | 70TUC17 | T/C | T/C | E2/E4 |
| 761 | 73TUC17 | T/T | C/C | E3/E3 |
| 762 | 75TUC17 | T/T | C/C | E3/E3 |
| 763 | 78TUC17 | T/T | C/C | E3/E3 |
| 764 | 83TUC17 | T/C | C/C | E3/E4 |
| 765 | 85TUC17 | T/C | C/C | E3/E4 |
| 766 | 87TUC17 | T/T | C/C | E3/E3 |
| 767 | 88TUC17 | T/T | C/C | E3/E3 |
| 768 | 89TUC17 | T/T | C/C | E3/E3 |
| 769 | 90TUC17 | T/T | C/C | E3/E3 |
| 770 | 92TUC17 | T/T | C/C | E3/E3 |
| 771 | 93TUC17 | T/T | T/C | E2/E3 |
| 772 | 94TUC17 | T/C | C/C | E3/E4 |
| 773 | 95TUC17 | T/T | C/C | E3/E3 |
| 774 | 96TUC17 | T/T | C/C | E3/E3 |
| 775 | 97TUC17 | T/T | C/C | E3/E3 |
| 776 | 98TUC17 | T/T | C/C | E3/E3 |
| 777 | 99TUC17 | T/T | T/C | E2/E3 |
| 778 | 101TUC17 | T/T | C/C | E3/E3 |
| 779 | 104TUC17 | T/C | C/C | E3/E4 |
| 780 | 108TUC17 | T/T | T/C | E2/E3 |
| 781 | 111TUC17 | T/C | C/C | E3/E4 |
| 782 | 113TUC17 | T/T | C/C | E3/E3 |
| 783 | 127TUC17 | T/T | C/C | E3/E3 |
| 784 | 132TUC17 | T/T | C/C | E3/E3 |
| 785 | 135TUC17 | T/T | C/C | E3/E3 |
| 786 | 138TUC17 | T/T | C/C | E3/E3 |
| 787 | 143TUC17 | T/C | C/C | E3/E4 |
| 788 | 144TUC17 | T/T | T/C | E2/E3 |
| 789 | 147TUC17 | T/T | C/C | E3/E3 |
| 790 | 149TUC17 | T/T | C/C | E3/E3 |
| 791 | 150TUC17 | T/C | C/C | E3/E4 |
| 792 | 154TUC17 | C/C | C/C | E4/E4 |
| 793 | 157TUC17 | T/T | C/C | E3/E3 |
| 794 | 164TUC17 | T/C | C/C | E3/E4 |
